# The Cost of Turnover in Home Healthcare: A Conceptual Model

**DOI:** 10.1177/08404704251412842

**Published:** 2026-02-06

**Authors:** K. A. P. Zagrodney, S. M. McKay, R. Jaffe, K. Myers, E. Sequera, D. Uzelac, E. C. King

**Affiliations:** 1 612784 VHA Home HealthCare, Toronto, Ontario, Canada; 2 Institute of Health Policy, Management and Evaluation, University of Toronto, Toronto, Ontario, Canada; 3 Ted Rogers School of Management, 120481 Toronto Metropolitan University, Ontario, Canada; 4 Department of Physical Therapy, University of Toronto, Ontario, Canada; 5 The Institute for Education Research, University Health Network, Toronto, Ontario, Canada; 6 Dalla Lana School of Public Health, University of Toronto, Toronto, Ontario, Canada

## Abstract

Despite the relatively high turnover rates and increasing demand for homecare, little is known about the costs of turnover for homecare employers. To create effective policies to reduce turnover and associated costs, employers require an understanding of what factors contribute to the costs of turnover. Building upon existing models from labour economic and human resources theories, we worked with subject matter experts from a large homecare employer organization to co-design the first known homecare cost of turnover model. The resulting model accounts for sector-specific cost differences and can be used to estimate the costs of homecare worker turnover. As homecare continues to face high workforce instability, clear estimates of the cost of turnover can guide organizations and decision-makers in the evidence-based design of retention policies and programs to the benefit of homecare workers, funders, those seeking homecare, and the broader health and social care system.

## Introduction

When a healthcare worker voluntarily leaves their job (i.e., turnover), this has consequences for those seeking care, remaining employees, employers, and funders. Within healthcare, homecare providers have some of the lowest retention rates,^1,2^ with annual turnover ranging from 44%-65% for homecare Personal Support Workers (PSWs)^3^ and 25%-31% for homecare nurses^4^ compared to 15%-44% for hospital nurses^5^ and 5%-7% for hospital physicians.^6^ There are multiple probable contributing factors to turnover for the mentally and physically demanding jobs of homecare PSWs and nurses, including lower wages, wider alternative job opportunities at similar wage levels, and structural precarity.^7,8^ With the rising dependence on homecare providers to deliver care to an ageing population,^9,10^ addressing high turnover in the sector will become increasingly critical to promote stability within care relationships, organizations, and the broader healthcare system. From an economic perspective, excessive turnover also creates avoidable costs for healthcare employers and funders. For instance, as high homecare provider turnover contributes to reduced capacity and care continuity in the sector, this can lead individuals to seek services in more expensive institution-based settings, such as hospitals.^10^ Hospital-based literature estimates the cost of nursing turnover to range from approximately $38,000-$140,000 (translated to 2025 CAD),^11-13^ but cost estimates specific to homecare are not yet available.

It is often difficult for employers to capture the costs of turnover due to the disparate organizational departments which are affected.^14,15^ However, quantification of these costs is necessary in order for employers to determine the cost-effectiveness of potential interventions to reduce turnover. Although such existing models from other occupations and sectors provide insight into factors which contribute to the costs of turnover, these models can miss context-specific costs. One of the most relevant models for healthcare turnover, Jones’ Nursing Turnover Cost Calculation Methodology (NTCCM), has been used in nursing literature^5^ and outlines types of costs to employers related to: filling vacancies (e.g., sub-contractors and overtime), recruitment (e.g., advertising and interviewing), hiring (e.g., employee processing), orientation (e.g., training and supplies), productivity (e.g., supervisor time), and exit from employment (e.g., loss of supplies and equipment).^16,17^ This model is clear and comprehensive but reflects an institution-based framing—some costs that would be incurred in a homecare setting are missing and some of the identified costs would not apply in a homecare context given the individual, visit-based nature of the work.

The nature of homecare work differs fundamentally from work in institutionalized settings in ways that are likely to affect the cost of turnover experienced by homecare employers. Not only are socio-demographics of the workforce significantly different between homecare and long-term care homes or hospitals,^18^ but job-related factors also significantly differ between sectors.^19^ Travelling between clients’ homes to deliver care, homecare providers tend to work more independently than healthcare workers in institutional hospital or long-term care settings, typically on pay-per-visits models (e.g., fee-for-service and hourly models). On a pay-per-visit model, changes in worker productivity, identified in NTCCM as contributors to turnover costs both early and late in the employment cycle, feature less prominently in homecare. Opportunity costs also look different in this context, where revenue is tied directly to the provision of units of service. Additional costs relate to the logistics of supporting a remote workforce, where resources such as cell phones and individual supplies of Personal Protective Equipment (PPE) may be more difficult to recover at the end of employment than they would be in an institutional setting. Such inherent differences associated with the homecare context result in different turnover-related cost inputs than in cost of turnover models which take an institution-based lens.

With the increasing reliance on homecare across healthcare systems in Canada and internationally^20^– both in response to peoples’ preferences to remain in their own communities^21^ and as a measure to manage healthcare costs,^10^ there is a heightened need to stabilize the workforce in this sector. Demand for homecare services is expected to increase by over 50% by 2031,^9^ which will compound current supply inadequacies.^22^ To promote workforce growth and address high rates of turnover, there is an urgent need for evidence-based retention policies for homecare providers.^23,24^ Policy-makers at the organizational and governmental levels require a clear understanding of the cost of turnover to enable them to evaluate the potential benefits of investments in workforce planning and sustainability. This model will enable policy-makers to compare these expected benefits to the costs of investing in retention strategies such as improved compensation packages or increased training and professional development opportunities.

### Objective

The objective of this study was to generate the first known conceptual model for capturing the cost of turnover for homecare providers at an employer level, with the goal of creating a model which can be used consistently in this sector. The availability of a standard model for calculating the cost of turnover would create an opportunity to compare costs across different jurisdictions and over time, as has been done for nursing with the NTCCM.^5^

## Methods

Building on existing models from other occupations and sectors, we co-developed a homecare cost of turnover model with subject matter experts. Collaborators included homecare leaders, human resource specialists, and researchers embedded within a large employer of publicly funded homecare providers in urban Ontario. They had expertise in homecare operations, human resources, business, and labour economics. Given the current lack of a cost of turnover model for homecare, inspiration was drawn from the NTCCM.^16^ The NTCCM pulls from human capital theory and human resource accounting models and is applicable to nursing in institutional settings. The NTCCM is comprehensive and has been applied across international nursing literature to capture the costs of turnover.^5^ Using the NTCCM as a starting point, we iteratively removed, added, and adjusted cost categories to better reflect the realities of cost of homecare provider turnover. Changes were made based on collaborator expertise and the broader turnover and homecare literature. The role of sub-contracted services in homecare underscored the need to feature these costs prominently in this model for homecare.^25^ Specific attention was also paid to updating the model to better reflect costs associated with contemporary human resources tools and practices (e.g., increased use of digital recruitment and management platforms^26^). Although the proportion of total costs in each category may vary across settings, the components of the model were designed to be generalizable to employers of pay-per-visit homecare providers across jurisdictions, including comparable Canadian and international organizations and across urban and rural settings.

## Results

**Figure 1.**
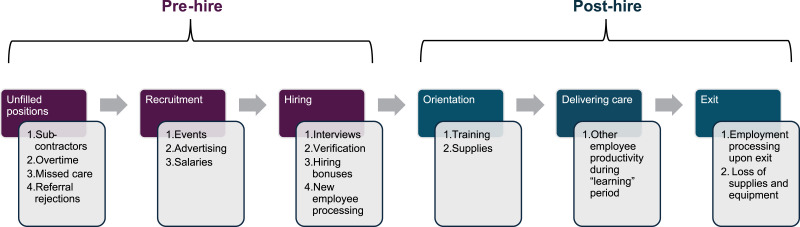
Employment cycle for a homecare provider from an employer’s perspective with associated cost of turnover categories

Through collaborative review of existing cost of turnover models and literature, we generated a homecare-specific model of turnover costs (Figure 1) which enumerates and separates contributors according to six overarching phases of the employee lifecycle, aligned with human resources workflows. Detailed within each phase are the major direct costs relevant to unfilled positions, recruitment, hiring, orientation, delivering care, and exit (Table 1). Other minor costs may also exist within each phase but would have a minimal impact on the overall cost of turnover.

**Table 1.** Homecare Cost of Turnover Model (HCTM)

	Cost examples
Pre-hire	
** Unfilled positions**	
Sub-contractors	- Higher pay to sub-contracted care providers and additional administrative burden to organize care delivery from a non-employee
Overtime	- Higher pay for overtime hours to existing employees
Missed care	- Opportunity costs from lost revenue streams from non-delivery; potential financial penalties for not providing care
Referral rejections	- Opportunity costs from lost revenue stream due to inability to accept new client referrals
** Recruitment**	
Events	- Event space, food and beverage, company-branded items
Advertising	- Personnel time and third-party payments for advertisements, social media, recruitment sites, and services
Salaries	- Personnel time for additional recruitment activities
** Hiring**	
Interviews	- Personnel time and administrative resources spent on screening and scheduling/conducting interviews
Verification	- Personnel time and third-party payments for background/criminal record checks and physical readiness tests
Hiring bonuses	- Bonuses to existing employees through referral programs
New employee processing	- Personnel time and administrative resources spent onboarding new hires across human resources, finance, and information technology departments
Post-hire	
** Orientation and training**	
Training	- Administrative support to organize training, physical space, virtual resources
	- Personnel time for both the trainee and trainer to participate in corporate and clinical new-hire training
	- Preceptor time to support new employee
Supplies	- Uniform, electronic devices, health, and safety materials
** Delivering care**	
Other employee productivity during “learning” periods	- Additional supervisor, scheduling coordinator, and peer coach/mentor time spent supporting new employees
** Exit**	
Employment processing upon exit	- Personnel time for offboarding, both directly and in digital platforms (e.g., termination of IT access, benefits, and pensions)
	- Costs for third-party services to aid in de-processing activities (e.g., survey platform and exit survey administration)
	- Personnel time to conduct exit interviews and analyze exit data
Loss of supplies and equipment	- Replacement of lost devices, health, and safety supplies

### Pre-Hire Cost Strategies

#### Unfilled Positions

If positions remain unfilled, capacity to provide service is reduced. Stopgap measures such as the use of sub-contractors and staff overtime may reduce the impact of this shortage; however, both represent more expensive ways to provide service. As overtime work is known to contribute to short- or long-term absences from work (e.g., due to increased sick days and burnout),^27^ use of this strategy may ultimately contribute to workforce unavailability. These consequences may be more difficult to attribute directly but should be recognized as a reason to use this strategy sparingly in addressing reduced capacity. Beyond the degree to which they can be addressed through overtime and sub-contracted providers, unfilled positions result in reduced capacity to provide service. In homecare, this can result in either individually cancelled visits (i.e., missed care) or an inability to accept new client referrals (i.e., referral rejections), both of which lead to opportunity costs, seen as reductions in revenue within the homecare pay-per-visit model.

#### Recruitment

Recruitment costs include costs to host events (e.g., event space and materials), advertising fees which frequently involve third-party digital platforms and service contracts, and the salaries of personnel executing tasks related to such recruitment activities.

#### Hiring

Screening and interviewing applicants include costs of personnel time associated with conducting the screening, personnel time on administration tasks pre- and post-interview, third-party payments for contracted services (e.g., applicant tracking system in Workday), costs to access physical/virtual spaces, and equipment expenses. Successful candidates who pass the interview stage often undergo verification processes (criminal record, physical readiness) which may require rental fees for physical spaces, digital/virtual platforms, personnel time, and third-party contractors to conduct the verification checks. Costs to set up new hires include administrative personnel time for tasks related to pay and scheduling, with information obtained filed in physical paper-based systems and/or the potential use of third-party business tools to aid in new-hire profiles. Employers may also pay for incentivization schemes, including sign-on bonuses for successful candidates and/or bonuses for referrals leading to a new hire. Although some of these recruitment activities will result in new employees, some resources will be spent on applicants who are ultimately not hired.

### Post-Hire Cost Categories

#### Orientation and Training

After the hiring process is complete, newly hired employees receive orientation related to corporate and clinical expectations, policies and practices, and training and equipment prior to delivering care. Training costs include the salaries of both the newly hired trainees and existing employees who deliver the training, as well as the cost for physical or virtual spaces used to conduct training. Preceptorships, wherein a new hire is accompanied on visits by an experienced employee, result in additional costs to the employer through preceptor pay. As part of supporting a remote workforce, homecare providers are typically supplied directly with their own supplies of personal protective equipment such as gloves, face masks, N95 respirators, gowns, boot covers, and anti-slip shoe grips. Employees may also receive uniforms, name tags/badges, cell phones, and transportation aids (e.g., a public transit card or company vehicle).

#### Delivering Care

Additional personnel time to support new hires is required from supervisors, schedule coordinators, and peer coaches/mentors as the homecare provider starts to build their client caseload and deliver care in the community. Continually supporting a team with high turnover may also contribute to indirect costs to employers via contributions to burnout of supporting staff members.

#### Exit

Upon termination of employment, an employer can incur additional costs related to formalizing the end of the employment relationship and recovering previously issued equipment. This includes administrative time spent formalizing the end of employment—directly with the employee, tracking and storing information in either physical paper-based filing and/or through digital human resources, information technology, and operational platforms. This may also include conducting and analyzing exit surveys/interviews (which can occur in-house or through third-party payments). Finally, there may be costs associated with lost equipment that might otherwise have been reissued to a new employee (e.g., if a company cell phone is not returned and the departing employee is not charged for this).

## Discussion

Understanding homecare provider turnover costs is critical for informing investments in retention to enable the required stabilization and growth within the increasingly relied upon homecare sector. Yet avoidable costs of excessive homecare provider turnover are not available in the current literature. To guide future cost calculations in this area, we co-developed the first known conceptual model specific to the cost of homecare provider turnover. While many costs associated with healthcare provider turnover are consistent across settings, this model considers impacts of unique characteristics of the homecare sector which differ from the institutional long-term care and hospital sectors. These include pay-per-visit models in relation to opportunity costs and worker productivity, and direct supply of equipment to providers travelling between homes (e.g., personal protective equipment and cell phones). This contemporary model also captures costs related to the use of digital platforms to manage and support a mobile workforce.

While the model builds on existing literature on the cost of turnover in healthcare (particularly Jones et al., 2006^16^), it deviates from existing models centred in institutional long-term care and hospital environments by accounting for unique characteristics that are central to the applicability in the homecare sector. First, the nature of costs related to unfilled positions experienced in homecare is different: homecare does not require a payment for “beds”^10^; therefore, unfilled positions in homecare do not translate to employer costs for “closed beds.”^5,17^ Instead, an inability to accept new client referrals due to low staff capacity translates into an opportunity cost for the homecare employer (i.e., missed potential revenue that could have been gained if the services were provided). Although many of the cost inputs for recruitment, hiring, and training are comparable across homecare and institution-based models, there are two key distinctions: (1) homecare providers are typically supplied directly with their own supplies of personal protective equipment, cell phones, and transportation aids and (2) this contemporary homecare model stresses costs associated with the use of digital platforms for supporting a remote workforce.

Beyond the logistical considerations, differences in the costs of turnover between institutionalized (e.g., hospitals and long-term care homes) and homecare sectors result from differences in the dominant payment models. Homecare providers typically function under a fee-for-service (pay-per-visit) model vs. institutionalized settings where providers are paid on a wage model, regardless of the number of tasks completed or the number of people who received care. Therefore, in homecare, lower productivity (fewer visits) near the beginning or end of employment are offset by corresponding lower payments to the pay-per-visit provider. Any further costs related to this lower productivity would instead be captured in the costs associated with “unfilled positions,” such as premium pay for overtime hours or sub-contractors to fill any gaps in care, or in opportunity costs that result from reduced capacity. Although the effects of reduced productivity of a pay-per-visit employee are somewhat mitigated, homecare employers do still need to account for reduced productivity of other workers supporting this individual, such as supervisors, schedule coordinators, and mentors. The cost of homecare provider turnover model outlined in this article accounts for these important sector distinctions.

### Limitations/Future Directions

This model was developed for employers of homecare providers at which both the organization and individual providers are compensated on a pay-per-visit model. The model was co-developed at a single, large, primarily urban homecare provider organization within the context of Ontario’s publicly funded homecare system. Although the components of the model were designed to be generalizable to employers of pay-per-visit homecare providers across jurisdictions, the proportions of total costs in each category may vary across settings. Development of a robust understanding of variations in the cost of turnover within the homecare sector will require applying this model to multiple environments, including Canadian and international contexts, as well as urban, rural and remote settings. Developed to calculate costs from an employer perspective, the model does not directly consider costs that may be incurred from other perspectives, such as the costs to providers themselves (e.g., lost income when job searching), to clients (e.g., potentially reduced access to services and care continuity), or to the broader healthcare system (e.g., through unnecessary expensive hospitalizations and extended alternative level of care hospital stays due to insufficient homecare capacity). The development of this model is an important first step towards understanding the cost of turnover in homecare.

## Conclusion

The homecare sector, which can expand rapidly without the need to create new institutional infrastructure, is well-placed to support health system capacity growth in response to growing needs.^10^ Currently, high turnover in this sector represents a significant barrier to unlocking the potential of homecare to increase system capacity. There is therefore a need for evidence-based, cost-effective initiatives to reduce turnover which requires an understanding of the costs of turnover in order to inform responsible investments. Homecare organization decision-makers can apply the co-designed homecare provider cost of turnover model put forth in this article to develop a better understanding of turnover costs at their organizations to inform retention strategies.
